# The Resurgence of Home-Based Primary Care Models in the United States

**DOI:** 10.3390/geriatrics3030041

**Published:** 2018-07-16

**Authors:** Mattan Schuchman, Mindy Fain, Thomas Cornwell

**Affiliations:** 1 Division of Geriatric Medicine and Gerontology, The Johns Hopkins School of Medicine, Baltimore, MD 21205, USA; mattan@jhmi.edu; 2 Division of Geriatrics, General Internal Medicine and Palliative Medicine, Arizona Center on Aging, Arizona Geriatric Workforce Enhancement Program, University of Arizona College of Medicine, Tucson, AZ 85724, USA; mfain@aging.arizona.edu; 3 Home Centered Care Institute, Schaumburg, IL 60173, USA

**Keywords:** home-based primary care, home health, house call, home care medicine, home based medical care

## Abstract

This article describes the forces behind the resurgence of home-based primary care (HBPC) in the United States and then details different HBPC models. Factors leading to the resurgence include an aging society, improved technology, an increased emphasis on home and community services, higher fee-for-service payments, and health care reform that rewards value over volume. The cost savings come principally from reduced institutional care in hospitals and skilled nursing facilities. HBPC targets the most complex and costliest patients in society. An interdisciplinary team best serves this high-need population. This remarkable care model provides immense provider satisfaction. HBPC models differ based on their mission, target population, geography, and revenue structure. Different missions include improved care, reduced costs, reduced readmissions, and teaching. Various payment structures include fee-for-service and value-based contracts such as Medicare Shared Savings Programs, Medicare capitation programs, or at-risk contracts. Future directions include home-based services such as hospital at home and the expansion of the home-based workforce. HBPC is an area that will continue to expand. In conclusion, HBPC has been shown to improve the quality of life of home-limited patients and their caregivers while reducing health care costs.

## 1. Introduction

Home-based primary care (HBPC) provides quality, patient-centered care for people underserved in the current healthcare paradigm where the patient must travel to the provider. Chronic medical conditions increase with age often leading to functional impairments that reduce the ability to access medical care. The ability to seek medical care is further diminished when a person has little social support and/or financial resources, such as family members to take them to appointments or funds to make accessibility modifications for their home. These access barriers lead to missed appointments, fragmented care, and poor control of chronic conditions. HBPC provides a way for patients with high-cost, complex, and function-limiting conditions to receive comprehensive, longitudinal primary medical and social care in their homes, and thereby avoid emergency room visits, acute hospitalizations, and institutionalizations. 

Besides filling a critical access gap, providing primary care in the home shifts the entire healthcare experience—for the better. The provider—a physician, nurse practitioner, or physician assistant—is visiting the patient on their own terms and on their own turf, changing the power dynamic of the encounter and facilitating relationship building; patients, caregivers, and providers often feel more connected. Operating in the home environment also gives the provider an opportunity to learn things that one could never discover in a typical clinic, such as the way a patient stores their medication (or not), the fall hazards a patient faces on their way to the bathroom, the empty refrigerator accounting for that involuntary weight loss, or the stress of a grandchild who had to quit her own job in order to serve as full-time caregiver.

## 2. Consider These Two Illustrative Cases

Case 1: Mrs. Helen Lupon is 83 years old and homebound from her multiple chronic conditions and functional impairments. Her 84-year-old husband, who is also her primary caregiver, is struggling with her care. In the year before enrolling in HBPC, she had 17 emergency department visits and 13 hospitalizations along with multiple inpatient stays for rehabilitation. Most hospitalizations were for heart failure with reduced ejection fraction, some for exacerbation of COPD, and one for a fall. The patient’s goals of care included not going back to the hospital if at all possible; after HBPC began, the patient required only one hospitalization over the next eight months. Home health was initiated along with telehealth. Medications were aggressively managed, and several were lowered or discontinued. The patient stabilized, home health was discontinued, and hospice services were brought in to assist the HBPC team and her family with her care. She died peacefully at home. Her quality of life during the last eight months was dramatically improved, and her husband voiced immense appreciation for all the help he received.

Case 2: Mr. Charles Turner is a fully ambulatory 65-year-old diabetic with concomitant mental illness and alcoholism. HBPC was medically necessary because he was poorly adherent to his diabetic regime, and over a 21-month period he had 44 emergency department visits and 27 hospitalizations (over half in the Intensive Care Unit for diabetic ketoacidosis). Frequent HBPC visits were made to engage the patient in his care, and behavioral health clinicians were consulted. New glasses were purchased which enabled patient to read his insulin syringes. After house calls were initiated, the patient’s adherence and quality of life dramatically improved, and he required only one hospitalization over the next two years.

HBPC programs are increasing in prevalence across the United States (U.S.). This growth is partly due to an interest among health systems in promoting value-based care. Though some solo practitioners provide house calls much as they were conducted 50 years ago, many new practices are based on an interdisciplinary team approach; this article will focus on this newer type of house call practice. Moreover, many practices ally with an entity that takes on financial risk for patient care and is rewarded with shared cost-savings resulting from better care. While HBPC practices take many forms based on their particular goals and business plans, these models are united by their focus on providing high quality, compassionate care in the home for those with the highest level of medical need and achieving cost savings. HBPC programs are increasingly recognized as adaptable and scalable, and value-based contracting with payors is becoming more common. This article describes the forces behind the resurgence of HBPC in the U.S. and then details different models including hospital based, government run, and free-standing programs.

## 3. From the Brink of Extinction

HBPC is making a comeback in the U.S. after a precipitous decline in the 20th century. While 40% of all U.S. medical care in 1930 occurred in the home [1], by 1950, this number dropped to 10%, and to only 0.6% of all patient encounters by 1980 [2]. The New England Journal of Medicine (NEJM) documented a further decline in their ominously entitled 1997 article, “House Calls to the Elderly—A Vanishing Practice among Physicians”. The NEJM review of 1993 Medicare (the U.S. government’s health care program for seniors and persons with disabling conditions) claims data found a further 30% drop in house calls between 1988 and 1993, from 1.6 million to 1.1 million, and down to only 984,000 house calls in 1996 [3].

The main reasons for the decline in HBPC were changing technology, fear of liability, and poor reimbursement. A stethoscope fitting into a black bag used to be the principle technology involved in making house calls. As medical technology such as lab equipment, X-rays, and ultrasounds became standard of care, it could no longer fit into a black bag and necessitated patients going to doctors’ offices or hospitals. Because the same level of technological care could not be done in the home, there was fear of increased liability. Finally, poor reimbursement discouraged house calls.

The decline in medical house calls has reversed in the last two decades, rebounding from under a million house calls in 1996 to 2.2 million house calls in 2016. There were an additional 3.2 million visits made to domiciliary facilities (Assisted Living Facilities, Group Homes) [4]. The change in course is due to overcoming the barriers for HBPC, as well as new factors that invigorated demand: increasing number of individuals with multiple chronic conditions and disability, federal legislation that allows people who would otherwise be institutionalized to remain at home, and an increasing focus on value-based care driven by Medicare.

## 4. Overcoming Barriers

Technology is no longer a major barrier. Electronic medical records allow access to patient charts virtually anywhere. Point of care testing for serum glucose levels and prothrombin international normalized ratios (PT/INR) are commonly done in the home and phlebotomy for any blood test can be done with the blood centrifuged in the car en route to the next house call. Portable lab instruments can perform tests such as a basic metabolic panel (BMP), brain natriuretic peptide (BNP), cardiac enzymes and blood gases with three drops of whole blood in as little as two minutes. Portable X-rays and ultrasounds services are now available in many areas and are also provided by house call companies. A smartphone can function as an electrocardiogram (EKG) machine and ultrasound console, as well as provide medical textbooks, drug databases, electronic medical records, medical apps, and can facilitate paperwork with remote scanning and printing. Modern technology brings quality medical care to the home; however, in some rural areas the technological barrier remains challenging.

Fear of liability has turned out to be unsubstantiated. A review of legal cases in the U.S. from the twentieth century found only two litigated law suits stemming from millions of house calls, with one of the two cases culminating in dismissal [5].

The financial barrier under Medicare’s standard fee-for-service (FFS) structure has not been completely overcome but has been significantly reduced. In 1996, house calls paid on average $3 more than an office visit with an average Medicare payment of $64. In 1998, Medicare added higher level house call codes and increased payments reflecting the increased complexity of care in the home. This change led to a 50% increase in house call payments with the most common house call visit payment rising to $95. House call payments have continued to increase so now most pay 100% more than in 1997 [6]. Medicare, in the past few years, has also added payments for services that are not covered under the typical visit fees, such as reimbursement for services provided when not face-to-face with a patient, preventative care, and for supervision of interdisciplinary teams and care plans. These additional payments reduce the financial barrier to making house calls. Medicare requires clinicians to justify the medical necessity for a house call rather than an office visit; however, Medicare grants HBPC practices some leeway in determining their own medical justifications and does not bind them to the Medicare definition of homebound (i.e., due to illness or injury a person must use an assistive device or aid of another person to leave their place of residence and have a normal inability to leave the home and leaving home requires a “considerable and taxing effort” [7]), as is required for Medicare Home Health (nurses and therapists who provide episodic services in the home). HBPC is also longitudinal and continues when patients are stable, in contrast to home health services which are intermittent and short term for an acute skilled need. The medical necessity cited by HBPC practices most often is that the patient is homebound and cannot get to an office. However, other reasonable justifications of medical necessity, such as frequent missed appointments, poor medication adherence, high utilization of the emergency department, or need to assess function in the home environment, are also acceptable.

## 5. A New Demand for Home-Based Primary Care

### 5.1. Demographics: The Aging of America

An increasing number of Americans could benefit from medically necessary house calls. In January 2011 the first baby boomers started turning 65, and 10,000 new boomers will join Medicare every day until 2029. The most rapidly growing age group in the U.S. is the 85 years of age and older, an age group projected to quadruple between 2000 and 2050 [8]. Approximately half this population needs assistance with at least one Activity of Daily Living (ADL); a quarter needs help with two, and one in six need help with three or more ADLs [9]. In 2011, 2 million individuals were homebound, and this number is expected to double over the next twenty years [10]. Despite these large numbers, the home-limited population is largely invisible to the health care system [11].

### 5.2. The U.S. Federal Rebalancing Legislation

Federal rebalancing legislation provides incentives for states to increase home- and community-based services that enable people to remain at home and reduce nursing home placement. The two main U.S. programs established under this initiative are Money Follows the Person [12] (MFP, which was authorized by the U.S. Congress in 2005) and the Balancing Incentive Program [13] (BIP, authorized in 2010). These programs provide financial and practical supports to enable patients to remain in their home or to transition from nursing homes back into the community. As of the end of 2015, over 63,000 people had enrolled in MFP across 46 states and Washington, D.C. [14]. Because of these and other programs, the percentage of government dollars for long-term services and supports going to home- and community-based services has leapt from 1% in 1983 to 55% in 2015 [15]. Efforts to helping nursing home-eligible people remain in the community has created an added demand for HBPC, as most of these individuals experience a significant level of functional impairment that make accessing care in an office setting difficult.

### 5.3. Health Care Reform

Health care reform in the U.S. is transforming reimbursement by incentivizing value over volume. By serving patients who are home-limited due to functional impairments, HBPC takes care of patients with the most complex and costliest conditions. These individuals are generally among the top 5% of patients whose care accounts for 50% of total expenditures. Hospitals are now penalized financially for excessive readmissions. Medical house calls have been shown to reduce hospital readmissions by 25–50%, thus reducing or eliminating readmission penalties [16]. Medicare has developed programs that share in cost savings produced by better care coordination and quality of care. One private multi-state HBPC program—which is exclusively dedicated to the home-limited population—generated over $59 million in shared savings in its first two years of participation in the Medicare Shared Savings Program ACO (2015/2016). This program cared for approximately 18,000 patients per year in 14 states. The year two savings were $44.5 million, yielding an average savings of $2500 per patient [17]. HBPC can also help reduce costs for at-risk contracts and other shared savings programs, such as Medicare capitation programs and bundled payment demonstrations.

### 5.4. Evidence for the Value of Medical House Calls

In two studies examining the value of house calls, Mary Naylor, Ph.D. (1999, 2004) demonstrated that nurse practitioner comprehensive discharge planning followed by house calls could cut health care costs in half and reduce 90 and 180-day readmissions by over 50% [16,18]. The Journal of the American Geriatric Society published two articles in October 2014 that significantly added to the evidence of the value of house calls. The first involved the Department of Veterans Affairs (VA, the U.S. government’s program to serve the health and other needs of military veterans) HBPC program. Costs were analyzed for nearly 7000 HBPC veterans dually enrolled in both the VA and Medicare in 2006. The authors found an overall 13.4% cost savings (over $6000 per veteran/year) with over 16% savings to the VA and over 10% savings to Medicare. The cost savings came principally from a 25% reduction in unnecessary hospitalizations. Analysis of 2007 VA HBPC patients found a nearly 60% reduction in hospital days, a 21% reduction in 30-day readmissions, and a nearly 90% reduction in VA nursing home days. Notably, the patient and caregiver satisfaction were the highest of any VA program [19]. HBPC’s marked decrease in nursing home days could also have a dramatic impact on Medicaid (the U.S. federal and state government health insurance program for individuals and families with qualifying low income), which in 2015 spent $71.5 billion (13% of its budget) on nursing home care [15].

The second article examined the costs of over 700 HBPC patients over two years at MedStar Washington Hospital Center in Washington, D.C. Patients receiving care from the team-based medical house call program had 17% lower Medicare costs than a group of over 2000 controls matched for medical complexity. This difference translates to over $8400 in savings per beneficiary in the study period and a total savings of $6.1 million. Hospitalizations were lowered by 9% and emergency department visits by 10%. The HBPC patients had more primary care visits, home health, and hospice service [20].

Independence at Home (IAH) is a three-year Medicare house call demonstration which drew upon the VA’s successful decades-long experience with HBPC. HBPCs participating in IAH receive an 80% portion of shared savings from Medicare after they reduce costs a minimum of 5%, meet established quality measures, and demonstrate high patient and caregiver satisfaction. Fifteen house call programs are currently involved in IAH. The program began with a yearly cap of 10,000 high-cost Medicare beneficiaries with two or more chronic conditions, two or more ADL deficiencies, and an emergent hospitalization and post-acute care service in the previous year. IAH has been one of the most successful Medicare demonstrations ever, with $32.8 million savings on over 15,000 beneficiaries (over $2000 savings per beneficiary per year) in the first two years. Data from subsequent years is still pending analysis. IAH beneficiaries had fewer 30-day readmissions, hospitalizations, and emergency department visits. Quality of care increased in all measured areas, such as follow-up within 48 h of hospitalization, medication reconciliation, and advanced care preferences documented [21]. In light of IAH’s success, the U.S. Congress has extended the demonstration from three to seven years and expanded the enrollment cap to 15,000 beneficiaries per year to allow for further regulatory and methodological refinements and for possible conversion to a new Medicare program.

### 5.5. Home-Based Primary Care Models

HBPC program models mainly differ based on two related factors: mission, which includes target population and geography served; and business plan, which includes payment structure and role within the health system. Examples of different missions include: longitudinal primary care for vulnerable, home-limited patients; improved end-of-life care; episodic home-based care to augment traditional primary care for at-risk patients; improved transitions of care with reduced readmissions; caring for a specific disease state; and teaching. Payment structures are typically either FFS (paid for each separate service performed) or value-based contracts (e.g., shared savings, at-risk contracts). Roles within the healthcare system span from hospital-based programs, to free-standing HBPC practices, to programs within integrated healthcare systems such as the VA. Many programs have multifaceted missions and incorporate a combination of different payment models.

Regardless of mission and business plan, most HBPC programs share a number of commonalities. Most HBPC programs include an interdisciplinary team, including nurses, social workers, other allied health professionals, and physicians, nurse practitioners, or physician assistants. The particular make-up of an interdisciplinary team varies depending on the program’s mission and ability to financially support team members. Although the VA has conducted extensive evaluations to determine optimal staffing and caseload ranges for their target population and system, no studies have been published depicting optimal program staffing for systems outside the VA.

Providers need to be skilled in primary care, geriatrics, and palliative care; core competencies include alignment of care with patient’s goals, ability to assess and manage complex chronic conditions and geriatric syndromes, provide family and caregiver support, access Durable Medical Equipment (DME) and community resources, manage polypharmacy, and provide symptom management and end-of-life care. HBPC programs often have annual mortality rates of 20–25%. Quality end-of-life care leads to most patients (>70%) dying at home, which far surpasses the national average of 33.5% [22]. Most HBPC programs have a clinical support staff to handle the large volume of calls from patients, caregivers, home health, hospice, and DME suppliers. The population also has significant mental health and community resource needs that benefit from the skills of a social worker. Social workers can be a part of the HBPC program or community based. Social workers have limited ability to bill under Medicare and are therefore difficult for many HBPC programs to afford. These programs can partner with social workers from home health agencies, social service agencies, or local non-profits. Another valuable discipline is a pharmacist to review complex medication lists and reduce polypharmacy. Pharmacists also cannot bill under Medicare, so programs often partner with health system or community pharmacists.

HBPC may support different populations depending on their mission and business plan. Clear identification of the targeted population is a key step in developing a successful HBPC program. The predominant population for most HBPC programs is the frail, home-limited elderly with multiple chronic conditions and functional impairments. The second population is younger, home-limited patients with neuromuscular disease such as Multiple Sclerosis, Amyotrophic Lateral Sclerosis, Traumatic Brain Injury, Cerebral Palsy, and Cervical Spine injuries with quadriplegia. A third group is comprised of high utilizers of the acute-care system who are not home-limited yet have difficulty engaging in the traditional healthcare system; some of these patients live with serious mental illness or other behavioral health problems that limits their access to care. Based on mission, programs can be tailored to serve a specific population, such as those dependent on ventilators, people with complicated diabetes, and people with sickle cell disease.

The final commonality among HBPC programs is dedication to a particular geographic area. Programs need to determine how large a radius they can cover, and if they will allow exceptions. FFS programs tend to limit geography to reduce uncompensated travel time. Service areas may be further subdivided for each day of the week to increase efficiency. Some programs increase efficiency by targeting Assisted Living Facilities, which enables providers to see multiple patients in the same facility on the same day. Programs with at-risk, value-based contracts will often travel greater distances to see patients, but in these circumstances the shared cost-savings that result from preventing avoidable hospitalizations more than cover the costs of the additional travel time. 

### 5.6. Hospital-Based HBPC Programs

Most hospital-based programs are located within academic centers, with fewer at community hospitals. Hospitalized HBPC patients are often costly to health systems. HBPC’s frail, complex patients often have high surgical risk, so symptoms such as chest pain and gastrointestinal bleeding are often treated medically or with “watchful waiting,” which reduces procedure revenue and increases length-of-stay. Health systems can benefit from downstream revenue from home health and hospice referrals. For example, the author’s (T.C.) two-physician HBPC practice in a Chicago-area academic center accounts for 7% of home health and over 12% of hospice referrals, resulting in over $300,000 of downstream profit for that center. This practice has also lowered hospital mortality rate by implementing palliative care principles. If a health system participates in at-risk contracts with an insurer, the HBPC program can reduce costs on the most expensive patients. Many hospital-based practices rely on a combination of FFS and alternative revenue sources. HBPC can also support hospitals in their efforts to reduce readmission penalties. Practices that focus on readmissions reduction commonly use an episodic HBPC approach to serve hospitalized patients stratified as high risk for readmission for 30–60 days to complement the services of their clinic-based primary care physician.

For academic institutions, HBPC offers a unique teaching opportunity. Its patient population is ideal for teaching geriatrics, transitions of care, home health, interdisciplinary collaboration, and palliative and end-of-life care to students of many different professions. HBPC gives trainees an opportunity to experience a high functioning interdisciplinary team in which each team member has an essential role and an equal voice in comprehensive care plan development. Home visits have been shown to fulfill all six of the Accreditation Council for Graduate Medical Education competencies [23] and relate to at least 16 of the 22 Family Medicine Milestone Project sub competencies [24].

### 5.7. VA HBPC Program

The VA runs the nation’s largest HBPC program with over 300 HBPC teams serving 59,000 veterans. The HBPC model began at a few large VA medical centers in the 1970s and has expanded throughout the US, Puerto Rico, Guam, and on Tribal lands. About one third of veterans who receive VA HBPC are in rural areas. The VA HBPC program provides comprehensive, interdisciplinary primary care, including behavioral health services, to veterans with serious chronic disease and disabling conditions [25]. Satisfaction surveys of VA HBPC recipients score highest of any VA program with over 80% of veterans rating satisfaction as 9 or 10 out of 10 [26]. Similar to other hospital-based programs, the VA HBPC reduces overall VA and Medicare health care costs through less hospital and nursing home utilization [19].

### 5.8. Free-Standing HBPC Programs

The structure of free-standing programs is significantly impacted by revenue sources. Programs that rely on FFS need to optimize billing and closely watch costs. Many FFS practices devote a large fraction of visits to assisted living facilities where a high concentration of patients increases efficiency. Most programs that rely on FFS need to find additional revenue sources, such as ancillary services, philanthropy, or value-based contracts, to break even.

Recognizing that by nature, HBPC is a low-volume, high-value service, some newer free-standing programs operate solely on value-based contracts. The contracts are typically with Medicare and Medicaid managed care contracts on a per-member-per-month basis. These programs take on some degree of financial risk for high cost populations. They offer a menu of services based on the complexity of the patient’s medical needs. Options may include office-based care, case management, home health, and for the most medically complex patients, HBPC. Some HBPC programs assume responsibility for the patient’s primary care, while for others the home-based medical and social care is adjunctive, collaborating with the patient’s primary care provider to coordinate care, improve patient/family education and self-care, and bringing in selected technologies (e.g., telehealth) to augment care in the home.

### 5.9. Future Directions

The next iteration of home-based care will provide services beyond primary care in a person’s home environment. One very promising model for potential replication is the Hospital at Home (HaH). HaH proposes that patients who meet certain criteria for hospitalization may safely be cared for at home with the proper allocation of resources. The many benefits to an in-home approach include reduction in overall cost and less risk of adverse iatrogenic outcomes, such as delirium, hospital-associated disability, and nosocomial infections [27,28,29]. A patient admitted to HaH is seen at home by a physician and nurse at least daily and has access to on-call physician coverage, with the patient or caregiver providing room and board [30]. Much like HBPC, the success of HaH in a financial sense is based in cost savings, rather than revenue generation. Thus, wider adoption has been seen in countries with single-payer healthcare systems, such as Australia and the UK. In the U.S., HaH has been implemented through the VA system for similar reasons. Implementation in Medicare managed care has also occurred with good results [31]. Pilot programs for HaH are emerging in innovative health systems that prioritize population health, and there has been movement towards developing a payment model for HaH in Medicare [32].

The U.S. is experiencing a national shortage of primary care providers and HBPC is only taught in a minority of medical, nurse practitioner, and physician assistant schools. Moreover, few providers are dedicated to a HBPC practice. Currently, only 1000 HBPC providers report making over 500 house calls annually in the United States [33]. Most of these high-volume providers are in urban or suburban areas; over ten rural states did not have any providers conducting over 500 visits, illustrating the severe shortage of HBPC in rural areas. Currently two national organizations, the American Academy of Home Care Medicine and the Home Centered Care Institute, are working together to grow HBPC and advance the workforce.

## 6. Conclusions

HBPC has been shown to dramatically improve the quality of life of home-limited patients and their caregivers. These programs also significantly lower health care costs by enabling patients to remain at home and avoid unnecessary emergency room visits, acute hospitalizations, and nursing home placement. This remarkable care model provides immense provider satisfaction [19]. Despite the improved outcomes and lower costs, HBPC has been slow to scale nationally, principally because Medicare does not provide adequate reimbursement. Work is ongoing to create value-based payments to make HBPC practices financially sustainable by rewarding programs for their quality and cost-savings. The structure of an HBPC program depends on specifics of its mission and business plan, but all include interdisciplinary team-based care and use the principles of geriatrics and palliative medicine to serve a high-risk population with complex medical and social issues. It is ever more imperative to support, refine, and expand HBPC as these models have demonstrated effectiveness in improving quality of care while reducing cost and supporting the needs of the growing number of home-limited people.
